# Own-name processing in toddlers with autism spectrum disorder: ERP evidence from an auditory oddball paradigm

**DOI:** 10.3389/fpsyt.2026.1780724

**Published:** 2026-04-27

**Authors:** Yige Wang, Yaru Zhang, Lin Zhang, Hongmei Tao, Ting Zhang, Zhi Shao

**Affiliations:** 1 Medical Research Center for Children with Autism in Chongqing, Rehabilitation and Treatment Center for Children with Autism in Chongqing, The Beibei Affiliated Hospital of Chongqing Medical University, Chongqing, China; 2 The Clinical Hospital of Chengdu Brain Science Institute, Key Laboratories of the Ministry of Education (MOE) Key Laboratory for Neuroinformation, University of Electronic Science and Technology of China, Chengdu, China; 3 Faculty of Psychology, Southwest University, Chongqing, China

**Keywords:** social auditory processing, autism spectrum disorder, ERPs, own-name processing, self-processing

## Abstract

**Introduction:**

Reduced responsiveness to hearing one’s own name is a core early behavioral predictor of autism spectrum disorder (ASD). This behavioral maker reflects atypicalities in self-related and social auditory processing, that are foundational to the development of social communication. To better understand the underlying neural mechanism of this alteration, the current study investigated neural correlates of auditory own-name processing in a sample of toddlers with ASD and typically developing (TD) controls using an auditory novelty oddball paradigm.

**Methods:**

Participants included 54 toddlers (30 ASD, 24 TD; 2-4 years) who passively listened to five auditory stimuli consisted of pure tones (500Hz and 1000Hz) and name types (self, familiar and stranger names). An auditory novelty oddball paradigm was utilized to assess neural response to standard and deviant stimuli, as well as to the different name types. Event-related potentials (ERPs) were recorded to measure neural activity during the stimulus processing.

**Results:**

Compared to TD controls, autistic toddlers showed reduced late MMN to deviant tones that was infrequently presented relative to standard tones; Analysis of ERP components during own-name processing revealed atypical neural response pattern: autistic toddlers exhibited enhanced central P3a coupled with decreased LDN amplitudes to one’s own-name relative to other names, and lack of parietal LPP effect typically observed in response to one’s own-name.

**Discussion:**

Our findings provide insight into the neural mechanisms underlying altered own-name processing in toddlers with ASD. These results suggest preserved early attentional capture of self-relevant salience, but atypical higher-order cognitive functioning during self-related and social auditory processing in toddlers with ASD.

## Introduction

Autism spectrum disorder (ASD) is a complex neurodevelopmental condition characterized by persistent challenges in social communication and social interaction, alongside restricted, repetitive patterns of behavior and interests (1). While prior research focused on early interpersonal abilities in ASD demonstrated marked difficulties in reciprocal social interaction, recent studies have begun to shift their focus on the self–other distinction underlying those difficulties. Among the early behavioral markers of ASD, atypical responses to hearing one’s own name, such as reduced attention orientation or delayed response to self-name, have been identified as a core indicator of atypical social responsiveness (2–5). Responding to hearing one’s own name is a fundamental form of social stimulation, which involves both basic acoustic discrimination and higher-order cognitive functions (e.g., self-identity recognition and social responsiveness), making it a critical window to explore the neural mechanisms underlying social cognitive problems in individuals with autism.

The ability to respond to one’s own name is a fundamental social–communicative milestone, typically emerging within the first year of life (6, 7). This response during infancy is crucial for not only developing social interaction skills, but also forming representations of the self and the other in cultivating abilities for self–other distinction—a core cognitive process enabling the differentiation of one’s own mental states from those of others (8). Prior research indicates that repeated failures to orienting to self-name during infancy is predictive for a later diagnosis with ASD and social functioning severity (3, 9–11). Furthermore, a longitudinal study reported that infants later diagnosed with ASD exhibited attenuated neural responses to one’s own name relative to an unfamiliar name as early as 14 months of age. This atypical neural pattern emerges prior to the onset of overt behavioral symptoms and is associated with poorer receptive language outcomes (12). These findings suggest that atypical self-name processing in early life reflects foundational difficulties in establishing the self–other boundary, which cascade into a broader challenge in social–cognitive functioning.

Event-related potentials (ERPs) enable the precise tracking of neural activities during name processing, revealing the time-course dynamics of cognitive operations that are inaccessible to behavioral measures alone. The P3 component encompasses P3a (or novelty P3) and P3b, where P3a primarily reflects early, stimulus-driven attention capture at frontocentral sites, whereas P3b reflects later, higher-order cognitive of self-referential processing at parietal sites (13). The late parietal positivity (LPP) component, indexing the late stage of self-referential processing, is also sensitive to self-name processing in neurotypical individuals (14, 15), whereas findings of this domain in autism are more variable. In the visual domain, ERP studies found comparable P3 amplitudes to seeing one’s own name compared to that of a familiar name in individuals with autism, while enhanced P3 responses to own name compared to all other names were observed in neurotypical individuals (16, 17), though both groups indicated a familiar effect (self and close other > stranger). In the auditory domain, absent enhancement of LPP amplitude for hearing one’s own name compared to other names was found in individuals with autism (18), with some revealed intact familiarity effect on an early N1 component (19, 20). Several studies have reported another ERP component that consists of a negative deflection following the early P2 amplitude, called the “subject’s own name” (SON) negativity, which is sensitive to own name relative to other names (19, 21). In addition to P3 and LPP components, distinct ERP components can be elicited by deviant or novel stimuli relative to repetitive standard stimuli using auditory oddball paradigms, such as the early mismatch negativity (MMN), which reflects the automatic detection of acoustic changes in the auditory environment (22), and the late MMN [also termed “late discriminative negativity” (LDN)], which indexes sustained auditory discrimination, stimulus classification, and the evaluation of salience (23). Previous analyses mainly focused on P3, SON negativity, and LPP components, which have often been reported to be related to the own-name processing.

To our knowledge, there are two studies that examined ERP correlates to hearing one’s own name in children with ASD. Only one study investigated toddlers aged 3 to 5 years old with and without a diagnosis of autism, indicating equivalent SON negativity-going ERP responses to own name compared with a non-sense stranger name in neurotypical children and children with autism, in which both groups showed stronger SON negativity to hearing one’s own name (self-name > a non-sense name), regardless of the familiarity of speakers (19). This component is often considered as an index of pre-attentive auditory detection of one’s own name (24, 25). However, no P3 and LPP components were found, and a familiar other name was not involved. A recent study investigated neural response to hearing one's own name in school-aged (7–13 years old) children with autism in comparison with neurotypical controls in an auditory oddball paradigm, revealing enhanced parietal P3 amplitudes for own name compared with a close other’s name, similar to what the neurotypical controls did (26). Together, influencing factors like modality of studies and age seem to have effects on the own-name processing in the autism population.

Despite the fact that certain aspects of self-processing may be atypical in individuals with autism, the findings are inconclusive. Several research found intact self-bias in perceptual and attention domains. For instance, individuals with autism showed enhanced performance (e.g., higher accuracy and faster RTs) for self-related items compared to other-related items (27–32), with some support from ERP studies showing intact own-name effect on attention in adults and children with autism (20, 33). Thus far, evidence supports the idea that altered self-bias in autism is not across domains (29, 32). Although lack of consistent own-name responsiveness was often reported in young children with autism, they may retain basic abilities for self-name detection, with atypicality in higher-order self-name processing (e.g., emotional and social meaning integration). Further lines of research investigating neural responses to one’s own name in toddlers with ASD are still warranted.

The aim of the current study was to investigate a time course of “early” automatic attention capture stage to “late” differentiated elaboration stage during self-name processing, in comparison with those of a familiar name and an unknown name. This was the first study that utilized the auditory oddball novelty paradigm to clarify the time course and neural basis of name-calling processing in a context of automatic attention orienting in a sample of toddlers with and without autism. Embedding own name and familiar and stranger names as novel among frequent standard and deviant stimuli (pure tones) allows us to measure from pre-attentive acoustic change detection (indexed by MMN), to involuntary attention capture (P3a), and to late-stage stimulus evaluation (LDN and LPP) during own-name processing.

## Methods

### Participants

Thirty toddlers with ASD (*M*_age_ = 43.05 months; *SD* = 6.95, range from 2 to 4 years old) and 24 chronological age-matched typically developing (TD) toddlers (*M*_age_ = 39.30 months; *SD* = 8.85) participated in this study. Children in the autism group were recruited from an autism rehabilitation center at a local hospital. All the children with autism met diagnostic criteria for ASD on the basis of DSM-5 criteria, and were diagnosed by a licensed clinician using the Autism Diagnostic Interview-Revised (ADI-R; 34) (Rutter) and the Childhood Autism Rating Scale (CARS; 35). Children in the autism group also underwent a medical assessment by a developmental pediatrician, and those with comorbid medical conditions were excluded. TD children without any psychiatric, current, or past neurological diagnosis; those without serious head injury; or those who had no first- or second-degree relatives with autism were included as controls. Participants in both groups had no visual or hearing problems reported by parents and therapists. This study was approved by the Beibei Affiliated Hospital of Chongqing Medical University. The children’s parents gave informed consent prior to the study, and participant’s personal identifying information (e.g., self-names) was kept confidential.

Data from 12 participants (7 with autism and 5 without autism) were excluded from analysis due to the following reasons: (1) insufficient number of artifact-free trials of EEG data; (2) participant non-compliance, e.g., refusal to wear the EEG electrode cap; and (3) persistent signal noise. The final sample includes 23 toddlers with autism (*M*_age_ = 40.76 months, *SD* = 7.52; CARS, *M ± SD* = 35.40 ± 1.89; ADI-R: mean Social, 24.30 ± 7.06; Communication, 15.2 ± 4.10; Restricted and Repetitive behaviors, 3.7 ± 1.29) and 19 toddler controls (*M*_age_ = 37.30 months, *SD* = 6.05).

### Stimuli and experimental design

The novelty oddball task consisted of 800 trials presented in four blocks of 200 trials each. Pure tones with 500 and 1,000 Hz lasting 50 ms were used respectively as standard (68%) and deviant (14%). Own, familiar, and stranger names were presented as novel stimuli (6% for each). Parents and therapists were asked to specify a familiar name that they frequently heard (e.g., a friend or classmate) and to choose a name that is most unfamiliar with the child from a list of five gender- and length-matched names. All the name stimuli were recorded by the same female speaker and normalized to a standard volume, and were digitized at 32 bit/44.1 kHz using the GoldWave program (Version 5, GoldWave Inc.). There was no significant difference in the duration of the name stimuli across name types and groups (*P*s > 0.73). Auditory stimuli were presented binaurally at 60 dB using earphones, while displaying images of a series of cars on the screen to maintain children’s attention without inducing restlessness.

Auditory stimuli were delivered in a pseudo-random sequence, adhering to three key constraints: (1) first five stimuli were standard sounds; (2) each deviant stimulus was preceded by at least three standard stimuli; and (3) each novel stimulus was preceded by a minimum of 10 standard or deviant stimuli. The presentation of novel stimuli (different types of names) was fully randomized across trials. To control for the confounding effect of visual stimulation on auditory name processing, identical images of cars were presented across own-name and other-name conditions throughout the task to maintain consistency. The inter-stimulus interval was fixed at 650 ms.

### Procedure

Upon arrival, the toddlers were asked to play in a child-friendly waiting area equipped with age-appropriate toys, and picture books, to reduce anxiety toward the experimental environment (room temperature: 22–24 °C; background noise < 40 dB). Concurrently, the parents received a detailed briefing on the study flow and strategies to support the toddler’s compliance (e.g., persuasion of cap-wearing and remaining still during recording). The parents were informed of the right to pause or terminate the experiment at any time.

During the formal experiment, the toddlers were seated on a chair for kids watching a cartoon clip during the preparation of the EEG cap. An EEG cap was placed over the scalp, adjusted to fit snugly but comfortably. Participants were seated in front of a computer monitor and were told in advance that they would hear some sounds while viewing images of various cars. They were instructed to stay as still as possible and would receive either a toy or a chocolate bar as the reward for completion. A passive oddball paradigm was used to elicit ERP responses to hearing one’s own name. If the toddler (particularly those with autism) showed signs of distress (such as crying or attempting to remove the electrode cap), the task was paused until the child became calm.

### EEG/ERP pre-processing and measures

Brain electrical activity was acquired using the Brain Vision Recorder software (36) using 64-channel elastic caps appropriately sized for the young children’s heads (Brain Products GmbH, Munich, Germany), with a sample rate of 500 Hz. The electrodes were positioned in accordance with the international 10–20 system, with the reference electrodes placed at FCz (frontocentral site) and the ground electrode placed at AFz (medial frontal site). Vertical electrooculogram (VEOG) was recorded using an electrode placed infraorbitally beneath the right eye. All scalp electrodes were initially referenced to the left and right mastoids, and electrode impedances were kept below 10 kΩ throughout data collection.

Offline analysis of the EEG data was conducted in Matlab R2014a using the EEGLAB toolbox (version 14.1.1b; 37). Raw data were digitally bandpass-filtered between 0.01 and 40 Hz and re-referenced to the average of the left and right mastoid recordings. Auditory data were segmented in epochs of 1,000 ms after stimulus onset with a 200-ms pre-stimulus baseline. Trials containing peak-to-peak amplitude deflections exceeding ±100 μV were excluded as artifact-contaminated. Independent component analysis (ICA) was applied to identify and remove stereotypical physiological artifacts (e.g., ocular movements). Additional visual inspection and amplitude-based artifact rejection were performed to ensure adequate removal of motion artifacts prior to further analysis. A trial was classified as invalid if artifacts were detected in more than 10% of the remaining electrodes, resulting in the exclusion of the entire trial. Only datasets with a minimum of 20 valid trials per experimental condition were retained for subsequent analyses. For each condition, ERP waveforms were averaged, re-referenced to a common average reference, and baseline-corrected per participant using the pre-stimulus (−200 to 0 ms) period. In the end, the grand average waveforms for ERPs were analyzed from 1,225 trials for the own name, 1,214 trials for the familiar name, and 1,230 trials for the unfamiliar name in the ASD group; and 1,136 trials for the own name, 1,155 trials for the familiar name, and 1,153 trials for the unfamiliar name in the TD group (*P*s = 1.000). Statistical analyses were conducted using R studio and SPSS 25.

The corresponding electrodes and time windows were selected based on the prior ERP evidence. The early and late MMN components (termed also as “LDN”, a deviant-specific component) were identified in the 200–300 ms and in the 400–550 ms time windows, respectively. The P3a and LDN components were identified during name processing in the 350–450 ms and 450–800 ms time windows all positioned at Cz, based on the visual inspection of the grand average ERP waveforms and their corresponding topography plot. The parietal LPP component was defined as the mean amplitude in the 650–800 ms time window positioned at Pz. Two-way mixed analyses of variance (ANOVAs) were performed for each component with pure tone/name types as a within-subject factor and group (autistic vs. neurotypical) as a between-subject factor.

## Results

### P3a analysis

**Figure 1:**
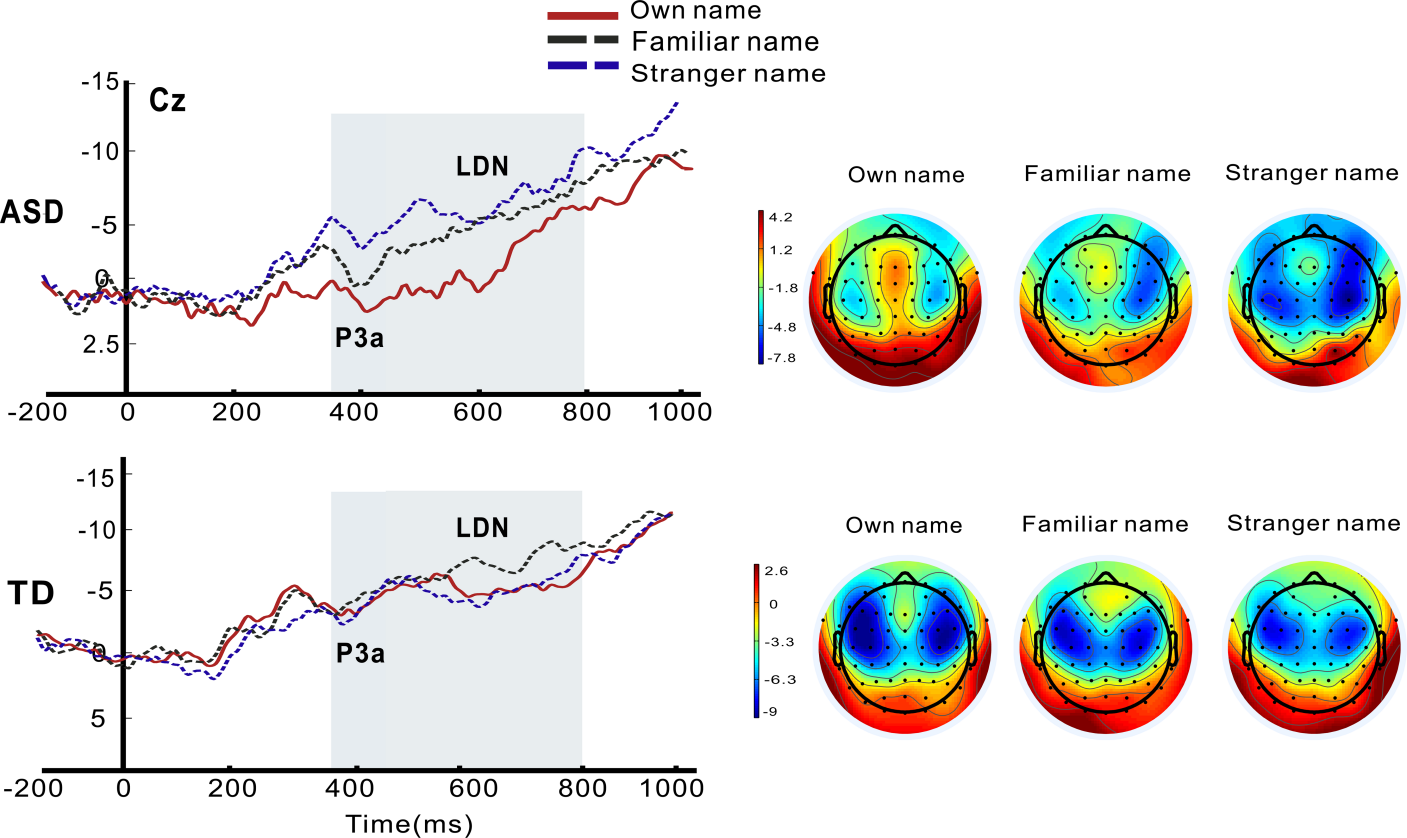
Grand average ERP waveforms for P3a and LDN components at Cz and topography plots (P3a, 350–450 ms; LDN, 450–800 ms) for autistic (ASD) and typically developing (TD) toddlers in response to hearing own name relative to familiar and stranger names.

A 2 × 3 repeated-measures ANOVA was conducted to examine P3a amplitudes over the central site, with Group (ASD vs. TD) as a between-subject factor and Name type (own, familiar, or stranger) as a within-subject factor. There was a significant main effect of Name type (*F*_2,80_ = 3.331, *p* = 0.041, *η*_p_^2^ = 0.077). Additionally, a significant interaction between Group and Name type was obtained (*F*_2,80_ = 4.742, *p* = 0.011, *η*_p_^2^ = 0.106). *Post-hoc* Bonferroni-corrected comparisons showed that a significant difference emerged between own name and a stranger name (*p* < 0.001), with a trend of larger P3a amplitudes for own name compared with a familiar name (*p* = 0.058) in the autism group (see **Figure 1**). However, similar P3a amplitudes were found across names in the TD control group.

### LDN analysis

For the LDN component, the ANOVA revealed a significant main effect of Name type (*F*_2,80_ = 4.130, *p* = 0.020, *η*_p_^2^ = 0.094), and a significant interaction between Group and Name type was observed (*F*_2,80_ = 4.264, *p* = 0.017, *η*_p_^2^ = 0.096). *Post-hoc* Bonferroni-corrected comparisons showed that while similar LDN amplitudes were found across names in the TD control group, weaker LDN amplitudes were observed for one’s own name compared with a familiar name (*p* < 0.05) and a stranger name (*p* < 0.01) for toddlers in the autism group (see **Figure 1**). Furthermore, toddlers with ASD exhibited reduced LDN amplitudes to own name (*p* < 0.05), but with comparable LDN to other names relative to TD controls.

### Parietal LPP analysis

**Figure 2:**
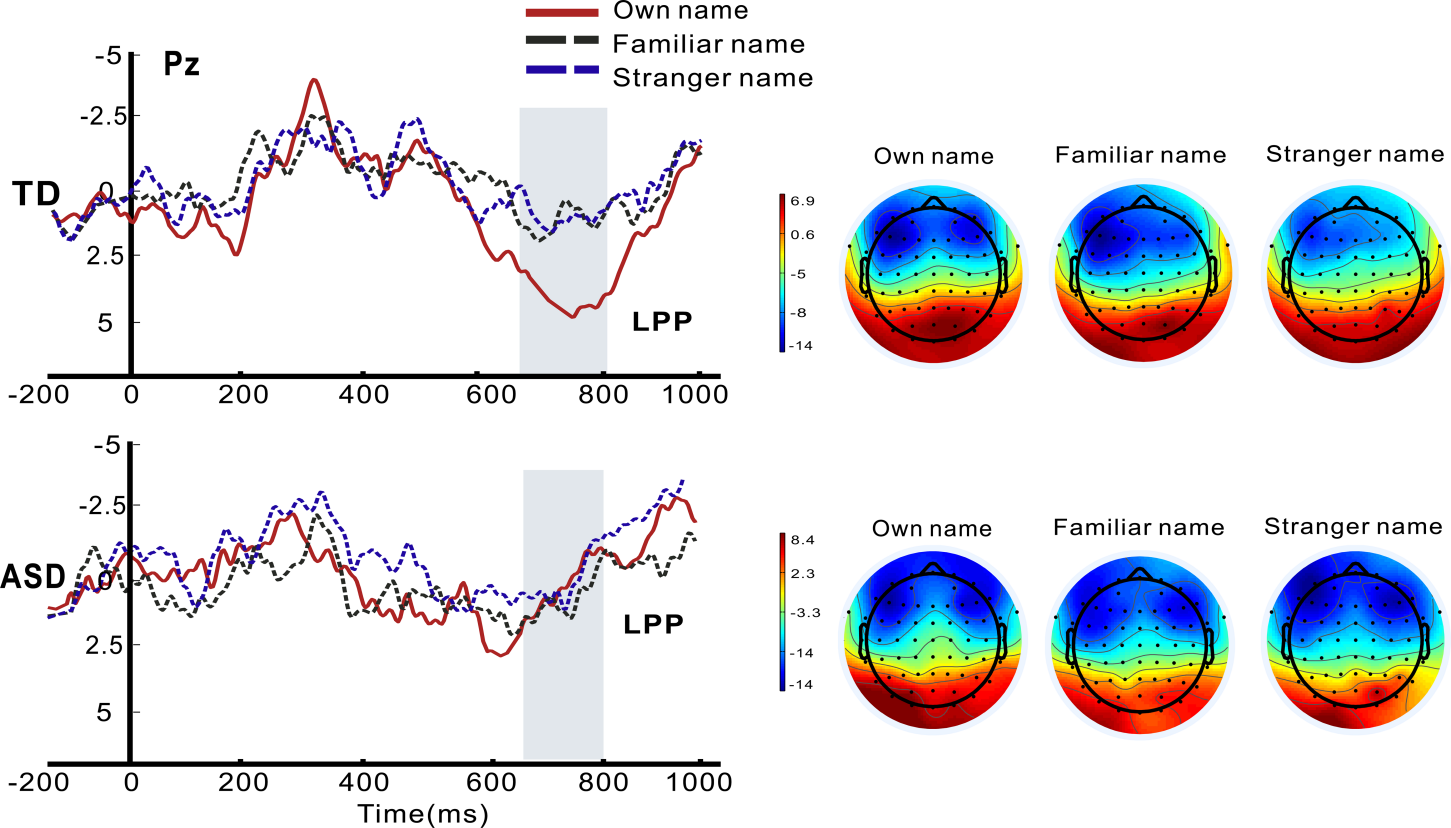
Grand average ERP waveforms of parietal LPP components and topography plots (LPP, 650–800 ms) for autistic (ASD) and typically developing (TD) toddlers in response to hearing own name compared with familiar and stranger names.

Repeated-measures ANOVA revealed a significant main effect of Name (*F*_2,80_ = 4.226, *p* = 0.018, *η*_p_^2^ = 0.096) with larger LPP amplitudes in response to own name compared to a stranger name. There was a significant interaction between Name and Group (*F*_2,80_ = 3.392, *p* = 0.039, *η*_p_^2^ = 0.780). *Post-hoc* Bonferroni-corrected comparisons indicated a significantly larger LPP to hearing one’s own name compared to a familiar name (*p* = 0.21) and a stranger name (*p* = 0.007) in healthy controls. However, toddlers in the autism group exhibited comparable neural responses across own, familiar, and stranger names. (see **Figure 2**).

### MMN analysis

**Figure 3:**
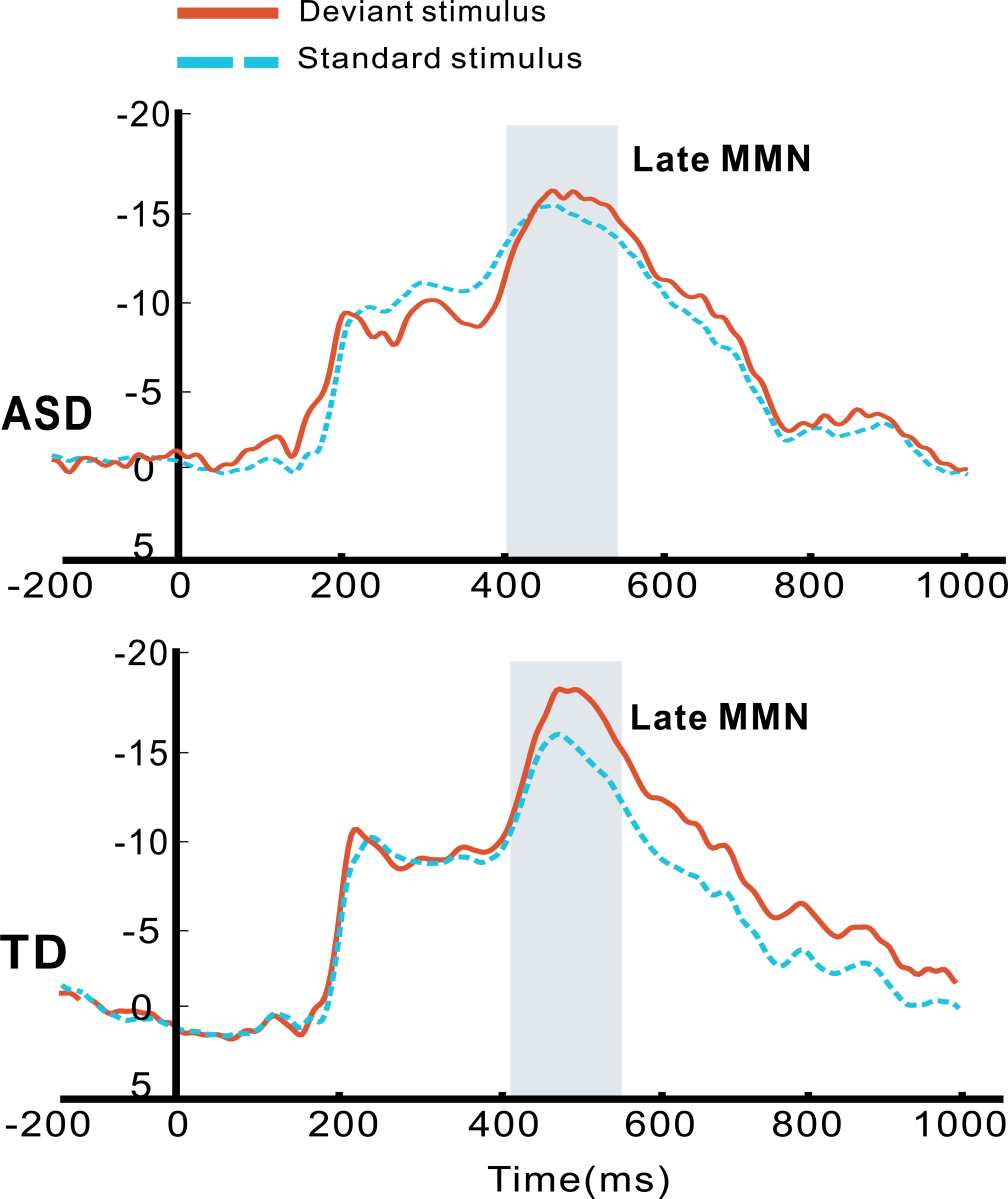
Grand average ERP waveforms of late MMN amplitude in the time window of 400–550 ms positioned at Cz, for autistic (ASD) and typically developing (TD) toddlers in response to standard and deviants that differ in frequency.

The novelty auditory oddball paradigm utilized presents a repetitive standard pure tone alongside infrequent deviant tones that differ in frequency. A 2 × 2 repeated-measures ANOVA was conducted to examine early and late MMN amplitudes, with Group (ASD vs. TD) as a between-subject factor and Tone (standard vs. deviant) as a within-subject factor. The results revealed a significant interaction between Tone and Group (*F*_1,40_ = 4.841, *p* = 0.034, *η*_p_^2^ = 0.108). While larger late MMN to frequency deviants were found in healthy toddlers (*p* < 0.001), blunted neural difference was observed between standard and deviant tones in toddlers with autism (*p* = 0.297), indicating poor acoustic discrimination. There were no main effects or interactions regarding early MMN amplitude across groups (see **Figure 3**).

## Discussion

The current study investigated neural correlates of auditory own-name processing using a novelty oddball paradigm in toddlers with autism and neurotypical controls, by means of ERPs. Core findings revealed enhanced P3a amplitudes at Cz, coupled with weaker LDN amplitudes to one’s own name relative to other names in toddlers in the autism group. Consistent with prior findings in adults with autism, larger parietal LPP amplitudes to own name relative to other names were observed in the control group, with no such effect found in the autism group. These results shed light on the distinct developmental trajectory of self-relevant and social auditory processing in early autism, highlighting dissociable neural mechanisms underlying early attention capture and social–semantic elaboration of self-relevant stimuli.

### Atypical P3a and LDN modulation during own-name processing in toddlers with ASD

We found that toddlers in the autism group exhibited increased P3a and reduced LDN amplitudes at central sites for own name versus other names. Names, as highly salient social stimuli, when presented as novel in an auditory oddball paradigm typically evoke a novelty P3 over central areas (38), reflecting the degree to which a stimulus captures and holds cognitive resources (13). In the current study, while TD toddlers equivalently elicit automatic attention capture toward any speech name as a salient social stimulus, toddlers with autism displayed strong involuntary orientation toward one’s own name, but not to other names (39). As it was also found in this study, the reduced late MMN to deviant tones suggest impaired pre-attentive acoustic change detection and altered context updating for non-salient acoustic stimuli. These results provide evidence for altered sensory processing and salience assignment in early autism (40, 41). The LDN reflects late-stage, sustained auditory discrimination and stimulus evaluation in auditory oddball paradigms, especially in infants and children (42, 43). We found that toddlers with autism appear to spare neural resources for self-relate stimuli, but engage in more prolonged, effortful, and resource-demanding late-stage discrimination for non-self-relevant stimuli. In TD controls, late evaluative processing remains stable across name stimuli due to their shared social and linguistic salience, regardless of familiarity. These results reflect intact early detection of the salience of their own name (20, 33, 44), together with heightened sensory sensitivity to ambiguous social input (45), as well as atypical social attention that was often observed in autism (46, 47).

However, according to the visual inspection of grand average ERP waveforms and topography plots, the observed P3a component elicited by one’s own name in toddlers with ASD was in the 350–450 ms interval, and weaker LDN negative-going amplitudes were in the following 450–800 ms interval; it is possible that the observed P3a may be superimposed on the LDN. The P3a is a frontal–central positive component (peaking 250–400 ms) linked to involuntary attention capture, novelty detection, and salience evaluation. The LDN is a frontal–central negative component (sustained 400–800 ms) reflecting acoustic discrimination and attention-related processes (48, 49). This topographical overlap introduces an alternative consideration: the enhanced P3a could alternatively reflect a weaker LDN during own-name processing in toddlers with ASD relative to TD controls, rather than a truly stronger P3a response. The results can be interpreted by atypical development of attentional engagement and sustained processing during social auditory stimulation, which is consistent with accounts of atypical orienting response to auditory stimuli and altered social attention in autism (2, 39).

This alternative interpretation carries important implications for understanding the nature of atypical self-processing in ASD. If the “enhanced” P3a is a product of a weaker LDN amplitude to own name, it would shift the focus from “enhanced early attention capture to one’s own name” to “inefficient late-stage social attention processing” as a core feature of own-name processing in toddlers with autism. This pattern stands in contrast to the more uniform and efficient automatic late-stage auditory processing of socially relevant stimuli (e.g., all names) found in TD toddlers, providing neurophysiological evidence for early emerging difference in how toddlers with autism process, evaluate, and attend to social auditory information that is less familiar.

### Altered parietal LPP response to hearing one’s own name in toddlers with ASD

Although previous studies on healthy individuals indicate larger parietal LPP (termed parietal P3 in some studies) responses to own name compared to other names across paradigms (21, 24, 50, 51), this component is not often investigated in existing studies that included young children with autism (12, 19), except for a recent ERP study that investigated children with autism in middle childhood (aged 4–12 years old) using an auditory oddball paradigm, which indicated larger parietal P3 amplitudes for own name compared with a close other name (Elkaddouri et al., 2025), despite the fact that only children with autism within the normal range of intellectual abilities were included.

As we initially hypothesized, we failed to find enhanced parietal LPP amplitudes for own name versus other names in toddlers with autism, consistent with previous evidence that the late stage of own-name processing is considered impaired (16–18, 52). The LPP, typically maximal at parietal sites, is widely recognized as a neural marker of late-stage cognitive processing, linked to emotional valence processing and self-referential elaboration (21, 53). It indexes the in-depth processing of the salience and social meaning of one’s own name. In TD toddlers, this elaboration is supported by a network of brain regions including the parietal cortex, temporoparietal junction (TPJ), and medial prefrontal cortex (mPFC)—regions critical for self-referential processing and social context integration (54), which has been reported to be atypical in autism studies using functional magnetic resonance imaging (55). Notably, atypicality in late stages of self-referential processing has been reported in studies of individuals with autism from different age groups (16, 17, 52, 56); thus, the absent LPP may not be a transient developmental delay, but a stable neural marker of diminished social–semantic elaboration of self-related stimulus.

Our findings suggest that, at least in early development, own name may retain exceptional salience and is capable of eliciting attentional engagement in autism. It may reflect a developmental specificity: early self-name processing may be supported by a relatively intact self-representation system, which later becomes increasingly intertwined with atypical social–semantic networks as development progresses (11, 29). Combined with a previously reported finding of enhanced P3a amplitudes at central sites, these results underscore a key dissociation for children with autism in the early developmental stage: while toddlers with autism can detect the salience of their own name, they fail to engage in a deeper social–semantic processing that gives the own name its unique self-referential and social–emotional significance (e.g., “this name is what my mum calls me when she hugs me”). The absent parietal LPP effect can be the result of the inability to integrate self-relevant stimuli to social–emotional contexts (47), combined with a failure of integration of the own name with self-identity, leading to context-inappropriate or absent behavior responses to one’s own name. Furthermore, it could also be due to atypicality in the development of early attentional network that may impact the higher-level social communication functions, such as selective attention and prioritization of social stimuli (39, 57). Our findings align with the proposal that the early stage of hearing one’s own name remains relatively intact, while late-stage processing is rather diminished (29, 55).

### Limitations and future directions

Some limitations should be considered. First, the potential superimposition of P3a and LDN complicates the interpretation of group difference in P3a amplitude, and advanced ERP analysis techniques or suitable experiment designs should be implemented to clarify the respective contribution. Second, a larger sample size of toddlers with autism with varying symptom severity is needed to replicate these findings before drawing solid conclusions. Third, the link between atypical ERP components and social behavior challenges remains unexplored, limiting the translational value of neural findings.

Future studies could also collect ERP recordings and concurrent behavioral data, such as eye-tracking and behavioral response to own name, to directly link the atypical neural response to their behavioral patterns. Additionally, longitudinal studies are essential to uncover the developmental trajectory of neural and behavioral patterns during own-name processing in children with autism. Finally, interventions targeting self-related processing and salience detection may help improve the development of social functioning in children with autism, and ERP measures could serve as objective biomarkers to evaluate early intervention efficacy.

## Conclusion

In sum, this study investigated the neural responses to hearing one’s own name in a sample of toddlers with autism and TD controls. We found that toddlers with autism exhibited atypical neural response during own-name processing, characterized by enhanced P3a coupled with weaker LDN amplitudes to one’s own name relative to other names, and a lack of parietal LPP effect during own-name processing that may reflect reduced integration of own name with a social context, as well as diminished self-referential processing that is foundational to early social development. Our findings demonstrate that atypicality of own-name processing spans multiple levels of auditory social attentional orienting and late-stage cognitive engagement, highlighting the complexity of self-related processing in autism.

## Data Availability

The data is not publicly available due to ethical restrictions protecting the privacy and confidentiality of research participants (children with ASD). Request to access the datasets should be directed to poppy.wang1989@hotmail.com.
